# Estimation of total glomerular number using an integrated disector method in embryonic and postnatal kidneys

**DOI:** 10.1186/2054-3581-1-12

**Published:** 2014-06-17

**Authors:** Michel G Arsenault, Yuan Miao, Kathleen Jones, David Sims, Jonathan Spears, Glenda M Wright, Sunny Hartwig

**Affiliations:** Department of Biomedical Sciences, Atlantic Veterinary College, University of Prince Edward Island, 550 University Avenue, Charlottetown, PE C1A 4P3 Canada; Department of Pathology and Microbiology, Atlantic Veterinary College, University of Prince Edward Island, 550 University Avenue, Charlottetown, PE C1A 4P3 Canada

**Keywords:** Nephron number, Disector, Nephron endowment, Kidney development, Kidney disease, Stereology

## Abstract

Congenital Anomalies of the Kidney and Urinary Tract (CAKUT) are a polymorphic group of clinical disorders comprising the major cause of renal failure in children. Included within CAKUT is a wide spectrum of developmental malformations ranging from renal agenesis, renal hypoplasia and renal dysplasia (maldifferentiation of renal tissue), each characterized by varying deficits in nephron number. First presented in the Brenner Hypothesis, low congenital nephron endowment is becoming recognized as an antecedent cause of adult-onset hypertension, a leading cause of coronary heart disease, stroke, and renal failure in North America. Genetic mouse models of impaired nephrogenesis and nephron endowment provide a critical framework for understanding the origins of human kidney disease. Current methods to quantitate nephron number include (i) acid maceration (ii) estimation of nephron number from a small number of tissue sections (iii) imaging modalities such as MRI and (iv) the gold standard physical disector/fractionator method. Despite its accuracy, the physical disector/fractionator method is rarely employed because it is labour-intensive, time-consuming and costly to perform. Consequently, less rigourous methods of nephron estimation are routinely employed by many laboratories. Here we present an updated, digitized version of the physical disector/fractionator method using free open source Fiji software, which we have termed the integrated disector method. This updated version of the gold standard modality accurately, rapidly and cost-effectively quantitates nephron number in embryonic and post-natal mouse kidneys, and can be easily adapted for stereological measurements in other organ systems.

**Electronic supplementary material:**

The online version of this article (doi:10.1186/2054-3581-1-12) contains supplementary material, which is available to authorized users.

## Abrégé

Les anomalies congénitales du rein et des voies urinaires (Congenital Anomalies of the Kidney and Urinary Tract, CAKUT) désignent un groupe polymorphe d’entités cliniques qui constitue la cause la plus fréquente d’insuffisance rénale chez l’enfant. Le CAKUT comprend aussi un grand nombre de malformations développementales, dont le syndrome de Potter, l’hypoplasie rénale, ainsi que la dysplasie rénale (maldifférentiation des tissus rénaux), toutes caractérisées par un déficit de néphrons. On reconnaît de plus en plus une masse néphronique congénitale réduite, d’abord présentée dans l’hypothèse de Brenner, comme une cause de l’hypertension chez l’adulte, de coronaropathie, d’AVC, et d’insuffisance rénale en Amérique du Nord. Les modèles génétiques de souris comportant une détérioration de la fonction rénale et de la masse néphronique fournissent un cadre pour permettre la compréhension de l’origine des néphropathies chez l’humain. Les méthodes actuelles de quantification des néphrons comprennent (i) la macération acide (ii) l’estimation du nombre de néphrons à partir d’une petite quantité de tissus sectionnés (iii) les modes d’imagerie tels que l’IRM et (iv) la technique de référence du disecteur et fractionnement. Malgré sa précision, cette dernière méthode n’est employée que rarement, puisqu’elle requiert main-d’œuvre, temps et argent. Par conséquent, plusieurs laboratoires emploient systématiquement des méthodes moins rigoureuses d’estimation du nombre de néphrons. Nous présentons ici une version mise à jour et numérisée de la technique du disecteur et fractionnement, que nous appelons la technique intégrée du disecteur, en utilisant Fiji, un logiciel ouvert et gratuit. Cette version mise à jour de la modalité de référence permet de quantifier les néphrons de manière précise, rapide et rentable dans les reins de souris à l’état embryonnaire ou postnatal, et peut aisément être adaptée aux mesures stéréologiques d’autres organes.

## Background

Defects in nephrogenesis and ureteric branching result in CAKUT. With an incidence of 1:400 [1, 2], CAKUT constitute one of the most frequent birth defects in humans, and the major cause of childhood renal failure [3, 4]. These pleiotrophic malformations comprise a multitude of renal phenotypes including renal agenesis, hypoplasia and renal dysplasia, associated with obstructive or refluxive anomalies of the ureter [5]. CAKUT are predicted to have different genetic origins [1], yet the molecular pathogenesis of CAKUT is not well understood. Nephron deficiency is a hallmark feature of CAKUT. In fact, nephron number varies greatly even within the normal distribution, ranging from 7,000 to 11,000 in mice [6], and from 200,000 to 1.8 million in humans [7]. Low nephron endowment within this normal range - though asymptomatic early in life, is associated with adult-onset hypertension [8–10], a disease affecting 73 million North Americans and a leading cause of coronary heart disease, stroke, and renal failure in North America [11, 12]. Despite the fundamental importance of nephron endowment to human health and disease, only a minority of molecular mechanisms underlying nephron morphogenesis have been identified. To this end, mouse models of impaired nephrogenesis and nephron endowment provide an important framework for understanding the molecular and developmental origins of human kidney disease.

Four main methods are currently employed to quantitate glomerular number (N_glom_) in the kidney [13]. The most accessible methods for estimating glomerular number by virtue of their ease of performance, relative low cost and rapidity, are (i) acid maceration and (ii) quantitation of glomeruli in a small number of histological sections. In the acid maceration method, the kidney is decapsulated, cut into small pieces and incubated in a weakly acidic solution. Tissue fragments are gently disrupted by manual aspiration which dissociates glomeruli in the suspension, and N_glom_ is counted within a known volume in a chamber similar to a hemocytometer [14, 15]. Acid maceration is a rapid, simple and inexpensive means of obtaining N_glom_; however, this method is subject to some of the same sources of counting errors and consequent low precision associated with hemocytometer use, including non-uniform suspensions and non-adherence to convention for counting glomeruli in contact with boundary lines or each other. A tacit assumption in the acid maceration method is that glomerular structural integrity is equivalent between sample groups. While not previously reported in the literature, it is feasible that disease states or genetic mutations that deleteriously affect the structural integrity of the glomerulus would render glomeruli more susceptible to acid digestion. Under such conditions, spurious differences in glomerular number could be obtained due to degradation of glomeruli in suspension, rather than true differences in glomerular number between groups.

A second commonly employed method involves evaluation of stained histological sections of the kidney. A small number of kidney tissue sections are chosen and glomerular number is counted and reported per area of tissue, or cortex. Although straightforward to perform, it is a biased sampling method that does not provide an accurate depiction of glomerular number for the entire kidney. Using this method, a glomerulus that is longer perpendicular to the plane of sectioning has a higher probability of being sampled in any given section than would the same glomerulus orientated parallel to the plane of sectioning. The number of glomeruli counted using this method not only depends on glomerular number and density, but also on their size and shape, which are not equally distributed throughout the kidney [16, 17].

The use of micro-magnetic resonance imaging (micro-MRI), also known as high-field MRI, is a third modality that can provide quantitative measurements *in vivo* and therefore holds great potential for future human applications. MRI provides both rapid and accurate evaluation of total glomerular number in healthy kidneys [18, 19]. However, access to the micro-MRI system is costly, rendering it less accessible to many laboratories. In addition, the accuracy of MRI is significantly reduced in kidneys with focal and segmental glomerulosclerosis [20], due to the low retention of the MRI contrasting agent cationic ferritin in the basement membranes of diseased glomeruli. Thus, disease states or genetic mutations that affect the integrity of the glomerular basement membrane may alter the retention of the contrasting agents in affected glomeruli, and can spuriously skew results.

The gold standard method for estimating N_glom_ is the unbiased physical disector/fractionator method. This stereological method involves exhaustively sectioning the kidney, and using a physical disector (a stereological probe) to count glomeruli in pairs of tissue sections. The application of stereology in biological systems and in the context of the kidney has been well-described [21, 22]. Briefly, this approach uses a pair of projection microscopes, a pencil and paper to manually circle and count glomeruli [23]. The physical disector method is extremely accurate because it is unbiased, but is very labour-intensive to perform, requiring a full day for an experienced technician to complete quantitation of a single rat kidney [13]. Commercially-available software packages offer powerful stereological alternatives to using projection microscopes for the physical disector/fractionator method [23], but are cost-prohibitive to most laboratories.

Members of the *Sry-Related HMG Box (Sox)* gene family have been shown to play master regulatory roles in a multitude of developmental processes including dorso-ventral patterning [24], stemness [25–27], male differentiation [28, 29], neurogenesis [30–32], cardiac outflow tract, B-lymphocyte [33] and spinal cord development [34], and skeletogenesis [35]. We have recently identified *Sox4* as a critical regulator of normal renal development *in vivo*[36]*.* Using an established *Six2-Cre* transgenic mouse line [37, 38] we ablated *Sox4* function in nephron progenitors and their cellular descendants (*Sox4*^*nephron-*^ mice). Using the acid maceration method, we demonstrated that *Sox4*^*nephron-*^ kidneys exhibit 48% reduction in nephron number compared to wild-type animals. In light of the low precision associated with this method and the inherent limitations associated with each of the existing methods of glomerular quantitation, we sought to develop an accurate, rapid and cost-effective alternative means of quantitating nephron number that could be of use to the nephrology community.

ImageJ is an open source image processing program through the NIH which has been freely available for more than 15 years [39]. Fiji is a free biological imaging package within ImageJ that comes pre-loaded with the TrakEM2 plugin for morphological data mining and three-dimensional modeling, and includes a stereological disector tool [40, 41]. By integrating the stereologic principles of the physical disector/fractionator method with automated light microscopy and ImageJ/Fiji software, we have developed an accurate, rapid, cost-effective method for quantitation of total nephron number in embryonic day (E)17.5 murine kidneys (including S-shaped bodies and maturing glomeruli), and estimation of nephron number in postnatal day (P)7 murine kidneys. Thus, we present the integrated disector method as a highly accessible adaptation of the physical disector fractionator method that can be easily adopted for in house application with minimal set up costs.

## Methods

### Mouse strains

The *Sox4* null (*Sox4*^*−*^) allele [33], conditional *Sox4* (*Sox4*^*C*^) allele [42] and *Six2-Cre BAC* transgenic line [37] have been previously characterized. All animal experiments were carried out in accordance with the policies of the Animal Care Committee at the University of Prince Edward Island and according to the Canadian Council on Animal Care guidelines.

### Histochemical staining

Kidneys were isolated from E17.5 and P7 *Six2Cre; Sox4*^*C/-*^*(Sox4*^*nephron-*^*)* and wild-type mice. Kidneys were fixed in 10% neutral buffered formalin, processed to paraffin blocks and exhaustively sectioned in the sagittal plane at 5 μm. Ribbons of serial E17.5 sections were mounted on glass slides, such that every section of the kidney was captured on a set of slides. Sections were stained with biotinylated peanut agglutinin (PNA) as described previously [43] with the following modifications: reduced incubation time of the biotinylated PNA to 10 min. and without hematoxylin counterstaining. Paired sections from P7 kidneys were selected using a systematic uniform random sampling approach as described [23]. Sections were stained with periodic acid-Schiff (PAS).

### Fiji processing of kidney section images

**Figure 1:**
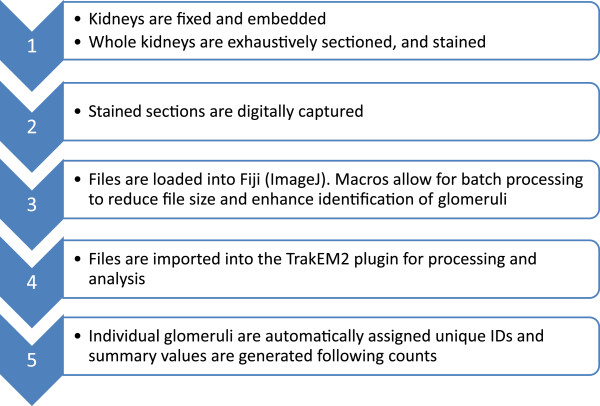
**Processing of kidney section images.** Schematic of workflow for quantitation of glomerular number using the integrated disector/fractionator method.

The established workflow for embryonic kidneys was as follows (Figure 1): Digital images of each stained section were captured by automated light microscopy using an Olympus BX61 Virtual Slide Microscope with motorized stage. Images were imported, converted to 8-bit grey and resized using Fiji software [41]. Additional file 1 (divided into three files due to size constraints) provide step-by-step video illustrations of the work flow. Briefly, all of the images for a single embryonic kidney were imported into a single stack and registered using the TrakEM2 plugin [40]. The disector tool within the TrakEM2 environment was then used to count every glomerulus within the kidney. Individual glomeruli were tagged with a unique ID which was maintained between sections, and reports were generated.

A similar approach was used to estimate glomerular number in postnatal kidneys. A pilot study was conducted to determine mean maximal glomerular size and the total number of sections through a P7 kidney, and the optimal section sampling fraction was determined to estimate total glomerular number (please see [43] for detailed protocol). Paired sections were imported, converted to 8-bit colour and manually aligned. The disector tool was then used to identify glomeruli that were either present on both paired sections or on only one of the two. Those present on only one of the two sections were counted as disector particles and used to estimate the total number of glomeruli (please refer to Additional file 2 for detailed instructions).

### Statistical methods

Two sample t-tests were performed using Minitab 16. Data are presented as mean ± standard deviation.

## Results and discussion

### Assessment of glomerular number in Sox4^nephron-^ kidneys

**Figure 2:**
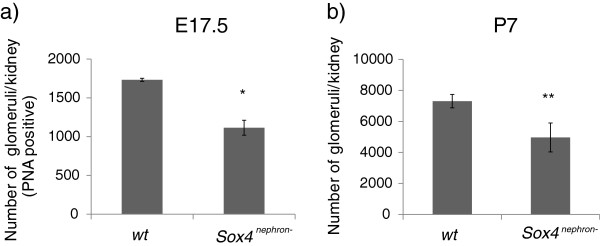
**Reduced nephron endowment in** ***Sox4*** ^***nephron-***^ **kidneys. (a)** Total glomerular number is significantly reduced in *Sox4* ^*nephron-*^ kidneys (36%) compared to wild-type littermates at E17.5 (***p = 0.001; wild-type n = 4; *Sox4* ^*neprhon-*^ n = 4). **(b)** Glomerular number is significantly reduced in a *Sox4* ^*nephron-*^ kidneys (32%) compared to wild-type littermates at P7 (*p = 0.012; wild-type n = 3; *Sox4* ^*neprhon-*^ n = 4).

Given the inherent limitations associated with existing methods of glomerular quantitation, we sought to develop an accurate, rapid and cost-effective alternative means of quantitating nephron number in the *Sox4*^*nephron-*^ kidneys. As the physical disector/fractionator method provides the most accurate quantitative measurements of glomerular number in both healthy and diseased kidneys, we sought ways to reduce the time and labour involved in this process, at low cost. To this end, we opted for automated microscopy combined with free open source Fiji image analysis software to develop a highly accessible workflow which we have termed the integrated disector method. The integrated disector method is a straightforward, rapid, and accurate means of quantitating nephron number in both embryonic and postnatal kidneys. In E17.5 mouse kidneys, PNA-stained S-shaped bodies and glomeruli are efficiently identified and counted using the TrakEM2 plugin and Fiji software. Quantitation of total glomerular number in E17.5 kidneys of *Sox4*^*nephron-*^ (n = 4) and wild-type (n = 4) mice demonstrated a 36% reduction in glomerular number (p = 0.001) in *Sox4*^*nephron-*^ kidneys compared to wild-type littermates (Figure 2a). In P7 kidneys, glomeruli were identified using PAS staining. A disector was used to obtain accurate unbiased estimates of total glomerular number from pairs of sections using the TrakEM2 plugin and Fiji software. Estimation of total glomerular number in P7 kidneys of *Sox4*^*nephron-*^ (n = 4) and wild-type mice (n = 3) demonstrated a 32% reduction in glomerular number in *Sox4-*deficient kidneys compared to wild-type (p = 0.012) (Figure 2b). The integrated disector method obtained glomerular numbers similar to those reported in the literature for both embryonic and postnatal wild-type kidneys [6].

Using acid maceration, we previously reported a substantially greater reduction (48%) in glomerular number in *Sox4-*deficient kidneys at P7. This difference is likely attributable to the high variability (low precision) inherent to the acid maceration technique. Variability in the acid maceration technique of glomerular quantitation is introduced by (i) pipetting errors (ii) non-uniform suspension of glomeruli and most significantly (iii) high standard deviation and its derivative coefficient of variability (CV) arising from the extrapolation of N_glom_ from a small volume (500 μL) to N_glom_ in the total volume (30 mL) [44]. Indeed, quantitation of N_glom_ using acid maceration is associated with a high CV in both wild-type kidneys (12.7%) and in *Sox4* mutant kidneys (15.6%), which may largely explain the difference in N_glom_ obtained using acid maceration vs. integrated disector methodologies. When restricted by cost and resources, acid maceration continues to provide useful insight into kidney development and physiology. This approach lends itself particularly well in instances where large between-group differences exist, as in the case of *Sox4-*mutant vs. wild-type glomerular number, and has recently been used for validation of new MRI approaches [19]. However, it is generally accepted and understood that stereology is a more robust approach for quantification of glomeruli. Indeed, the high variability inherent to the acid maceration technique was a significant motivation for us to implement cost-effective in house stereological methods for glomerular quantitation.

### The integrated disector method is cost-effective and accessible

Several open source biomedical image analysis software packages have recently become available including Endrov [45], Icy [46], BioImagXD [47] and Fiji [41]. Each package has a specialized focus and toolset to facilitate processing of complex biological spatio-temporal image data obtained mainly by microscopy. Certainly, multiple strategies can be developed to perform glomerular quantitation with high accuracy, speed and economy. However, to the best of our knowledge, we are the first to report a means of combining the high accuracy of the physical disector/fractionator gold standard method with the power and economy of open source image analysis software.

Although many groups are studying the effects of genes and various environmental factors on the development and function of the kidney, there is currently no broadly accessible method for accurately determining if there is an effect on nephron endowment. In the present study, we have optimized PNA and PAS staining of histological sections to identify glomeruli in embryonic and postnatal kidneys. We have updated the physical disector/fractionator method by combining semi-automated microscopy image capture with Fiji/TrakEM2 to streamline data collection and image analysis. With minimal setup costs, these tools permit accurate, efficient and rapid stereological measurement of glomerular number in many laboratories, and can be applied from early embryonic development through to the adult mouse. Implementing the integrated disector requires laboratories to follow the same steps associated with the physical disector/fractionator method from tissue embedding to exhaustive sectioning and staining of tissue [23]. The power of our approach is in its ease of set up and accessibility for counting N_glom_ using a freely available disector tool which allows for unbiased estimation of N_glom_. Total time for image acquisition and analysis for an experienced user and full automation would average 2-3 hours per kidney. Smaller laboratories that may not have access to motorized microscopes can easily outsource the image capture component to collaborators while performing the Fiji/TrakEM2 quantitation in-house. Alternatively, researchers can manually capture overlapping partial images of serial sections at the appropriate magnification, and sections can be stitched using the Grid/Collection stitching plugin built-in to Fiji. Stitched sections can then be loaded into the TrakEM2 environment and analysed as described. Other refinements and adaptations can be performed to best suit the needs of individual research teams, including the acquisition of volumetric data, and can be broadly applied towards stereological measurements in other organ systems.

## Conclusions

In summary, we have adapted the physical disector/fractionator method of stereological glomerular quantitation using semi-automated microscopy together with open source Fiji software and the TrakEM2 plugin. We present the integrated disector method in its entirety through step-wise video and screen capture, in the hopes of offering the general nephrology community an accurate, rapid, cost-effective and therefore highly-accessible means of glomerular quantification in embryonic and postnatal kidneys.

## Electronic supplementary material

Additional file 1: **Stepwise guide to using TrakEM2 for glomerular quantitation.** **1.1.** Step-wise AVI video screen capture guide for setting up disectors in the TrakEM2 environment. **1.2.** Step-wise AVI video screen capture guide for importing the image stack and manually aligning the kidney pair using TrakEM2. **1.3.** Stepwise AVI video screen capture guide for tagging glomeruli using the disector tool and generating the report in TrakEM2. (ZIP 22 MB)

Additional file 2: **General outline for performing disector counts using TrakEM2.** (PDF 1 MB)
