# Supplementary material for: Estimation of total glomerular number using an integrated disector method in embryonic and postnatal kidneys
Source: Can J Kidney Health Dis. 2014 Jun 17;1:12. doi: 10.1186/2054-3581-1-12 (PMC4349599; doi:10.1186/2054-3581-1-12)
Supplement: Supplementary file 2 — Additional file 2: General outline for performing disector counts using TrakEM2. (PDF 1 MB) [file 40697_2014_11_MOESM2_ESM.pdf]

Arsenault et al. 2014

Additional file 2 – General steps for disector counts using TrakEM2

1. Import images as a single stack
    a. File> Import > Image Sequence…

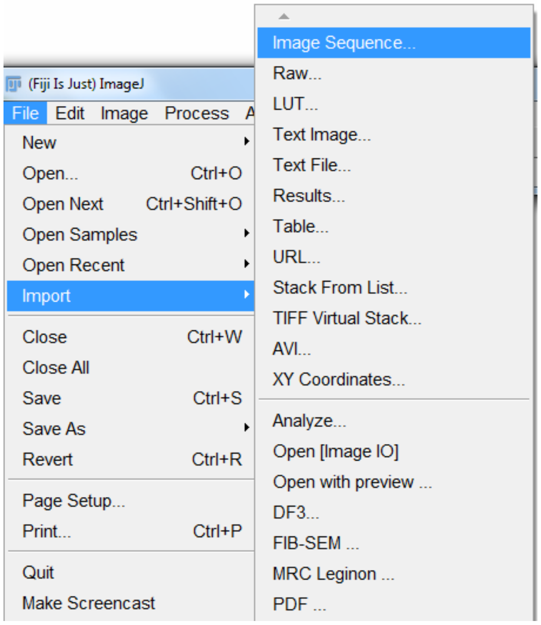

    b. Select folder with reference and lookup sequence and select "Use virtual Stack" in order to save on memory.

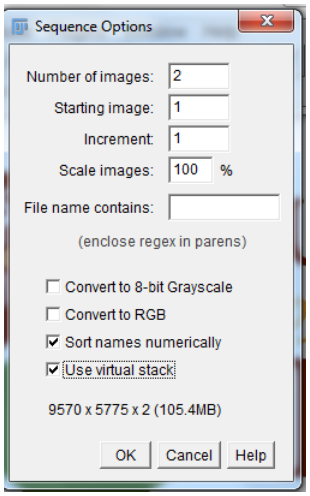

2. Open TrakEM2 environment
    a. File> New> TrakEM2 (blank)
       *later a template can be created to save some time and the TrakEM2 (from template) option can be used *

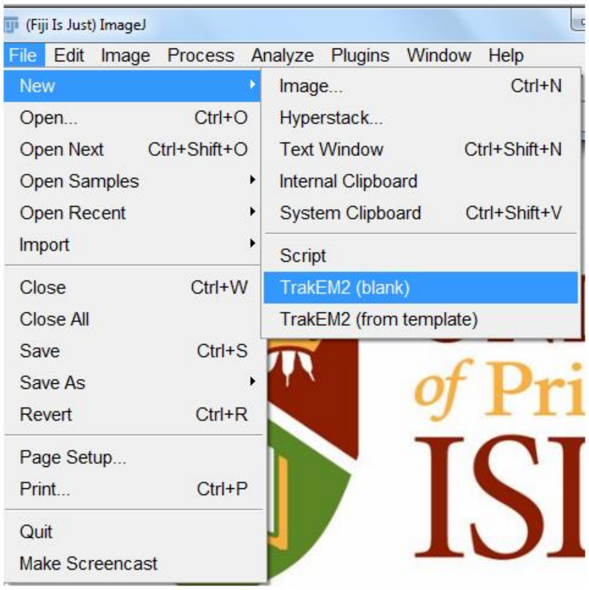

3. Set up the Template and Project Objects (dissector, area_lists, etc.)
    a. Create a template with the items to be used in the TrakEM2 project

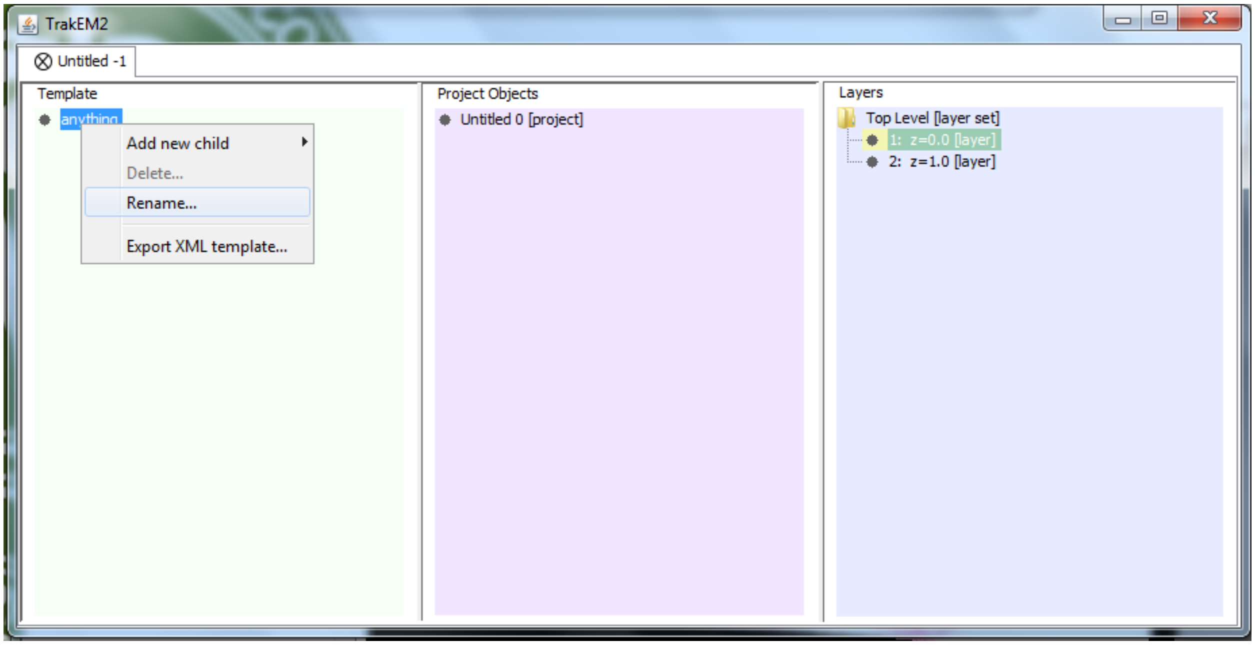

b. Create child nodes for each of the object categories

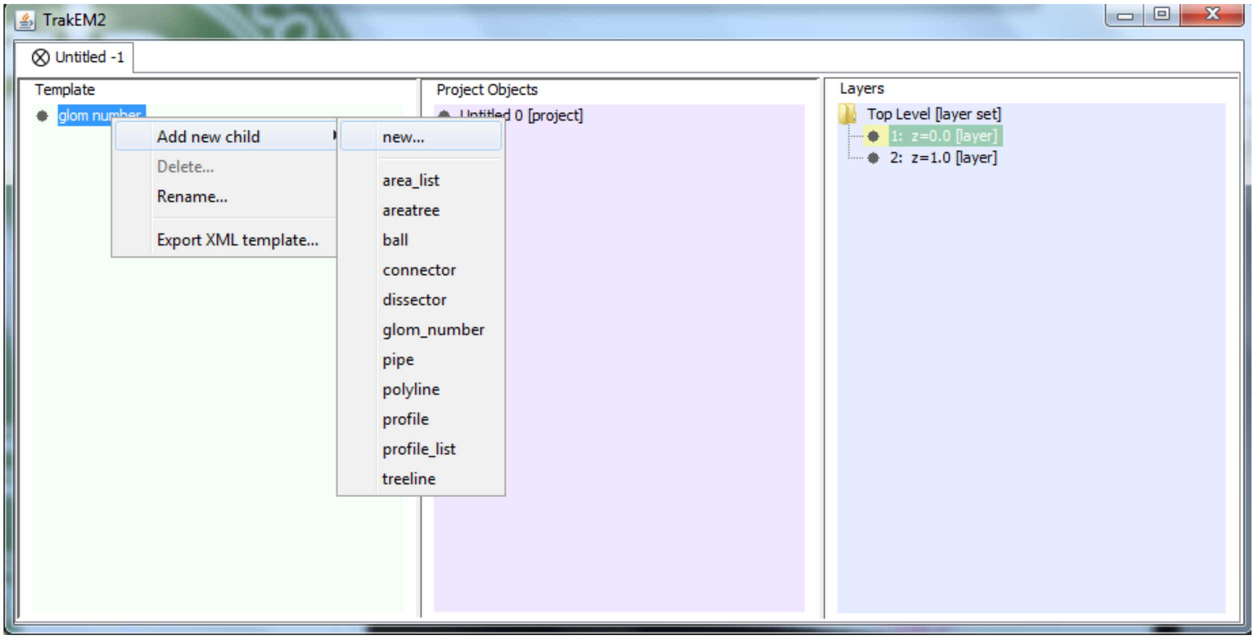

c. Add a disector to each object category

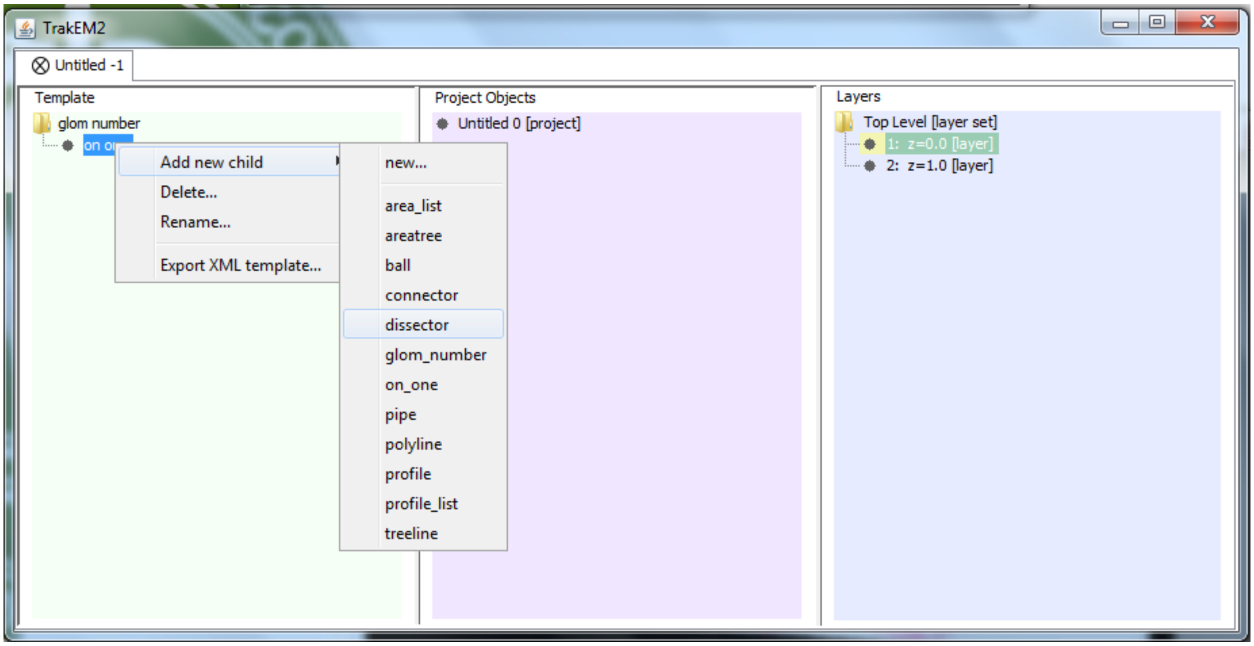

d. Drag and drop items from the “Template” area to the “Project Objects” area

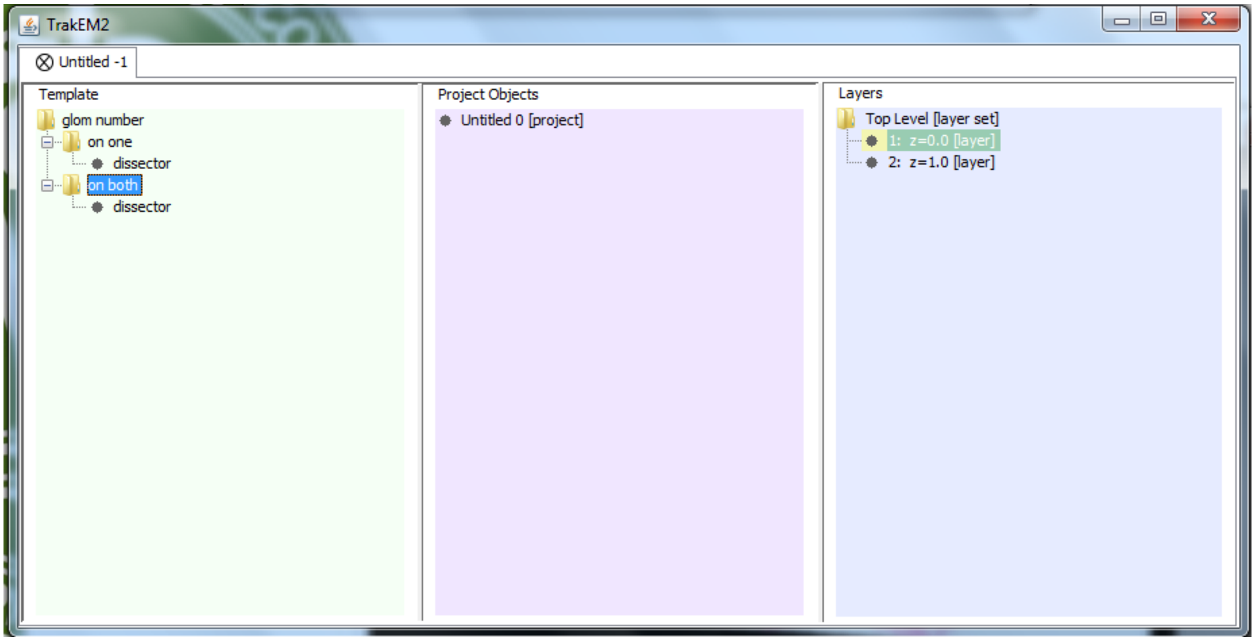

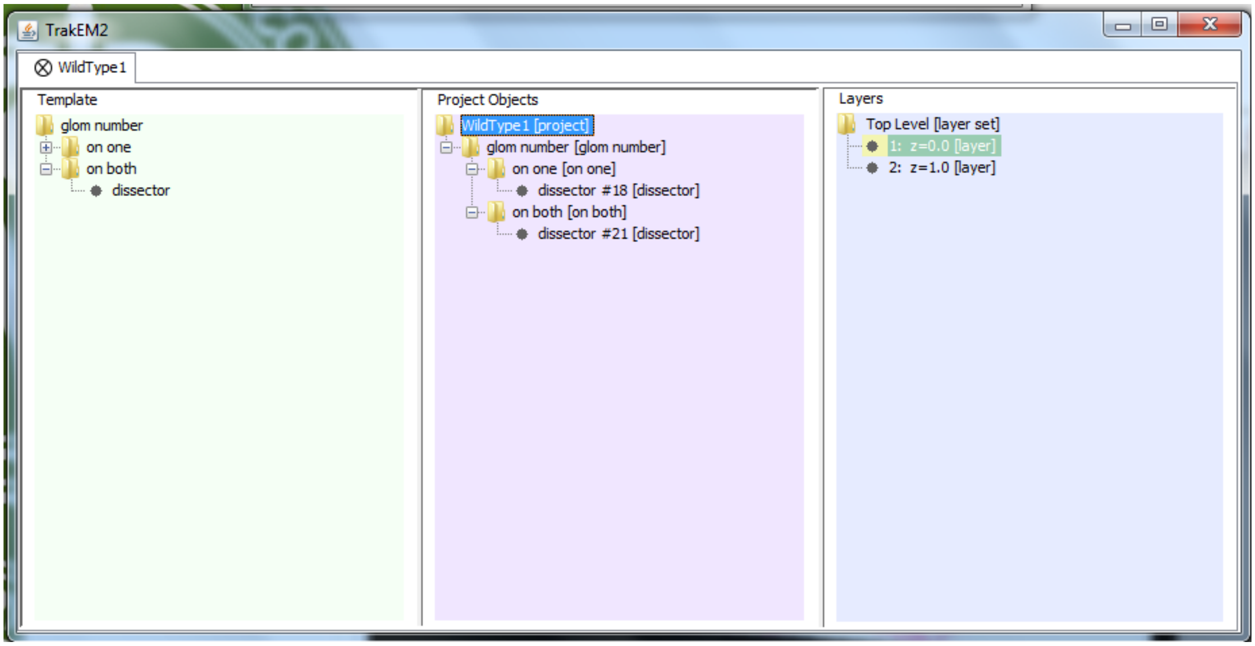

e. Disectors can be renamed to distinguish them in the TrakEM2 environment

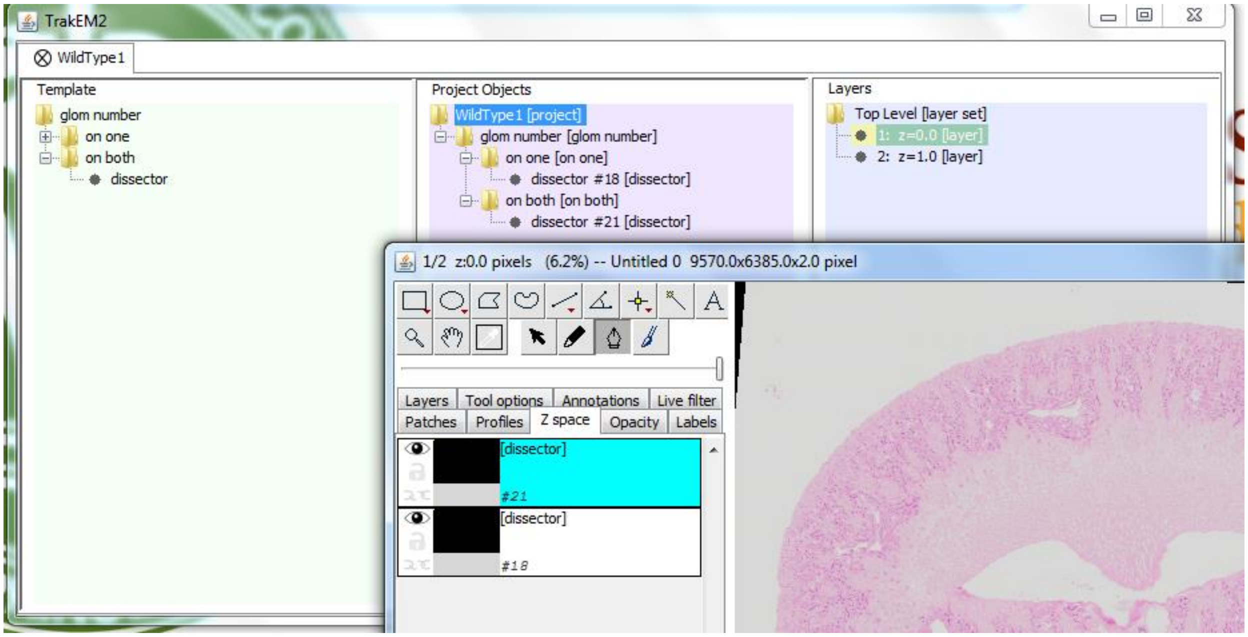

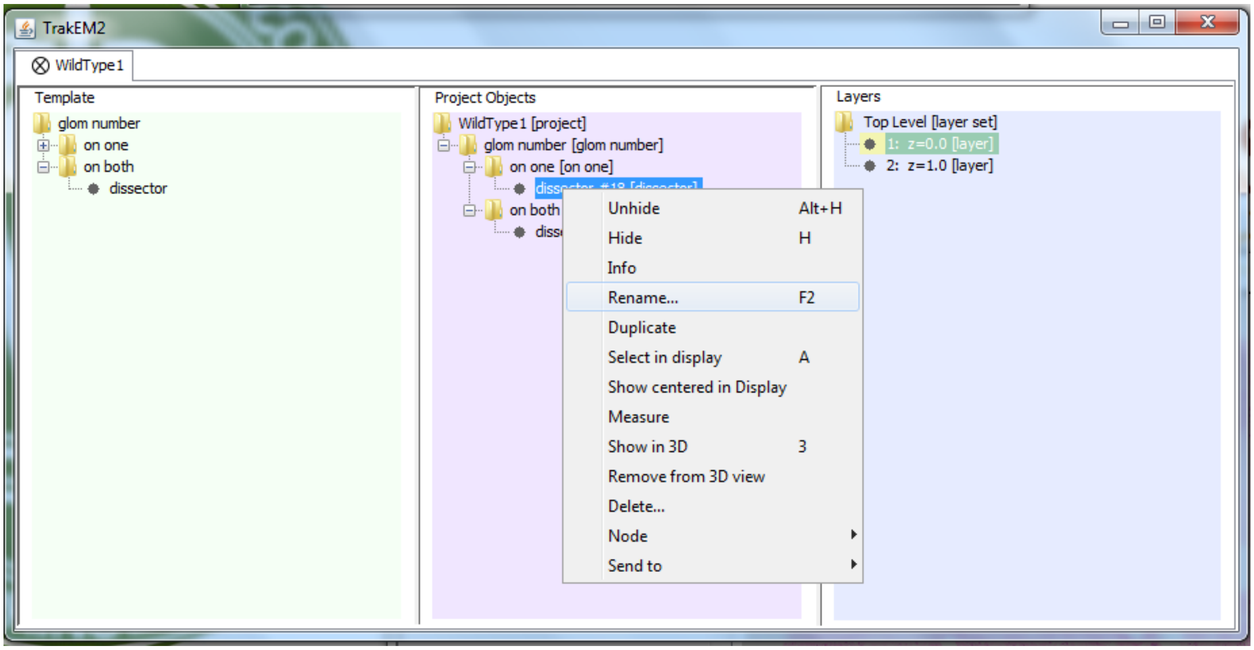

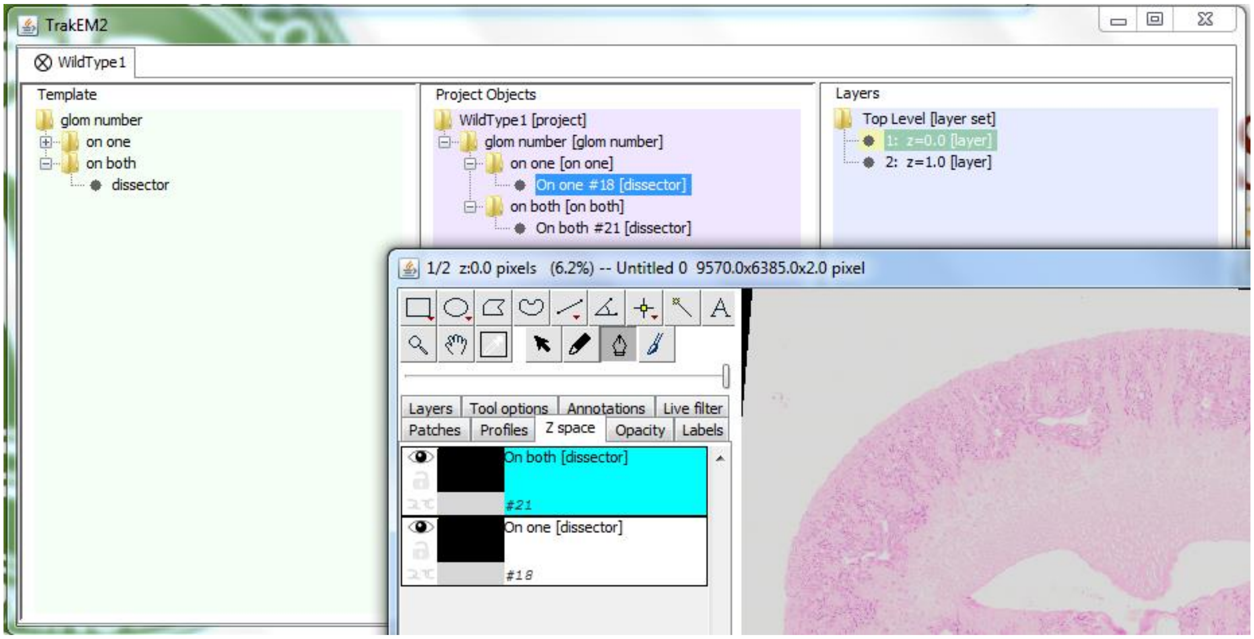

4. Import the image sequence into TrakEM2

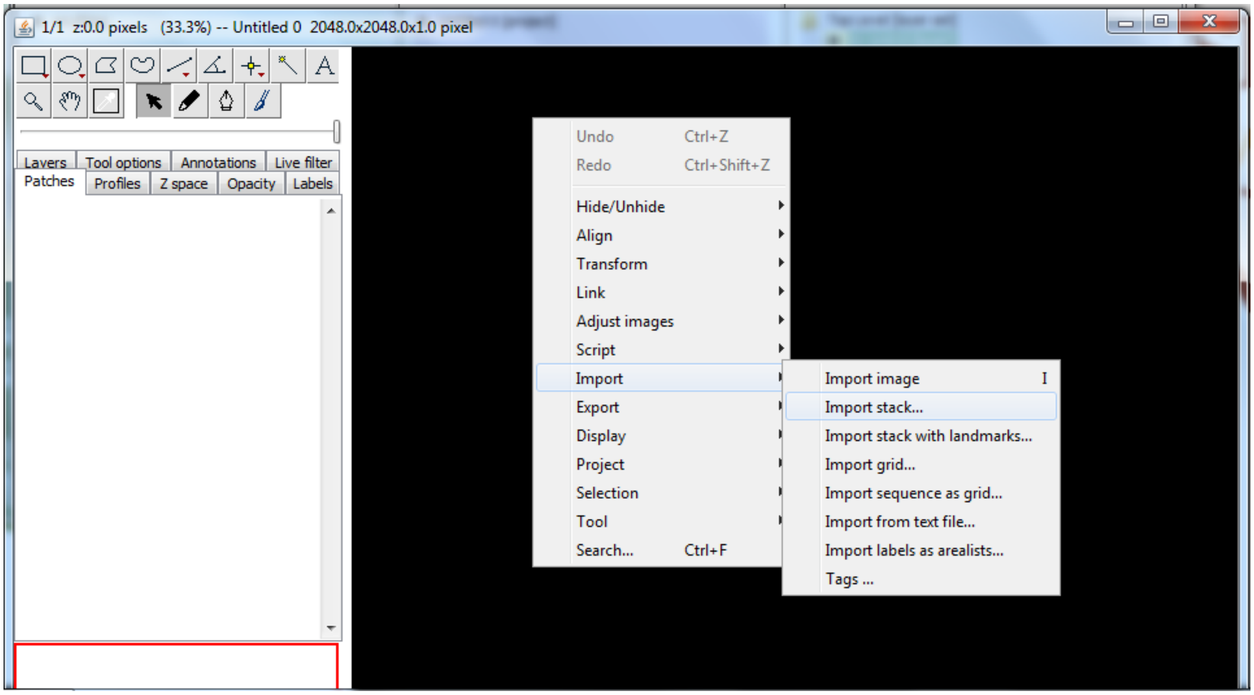

5. Align the layers manually or automatically

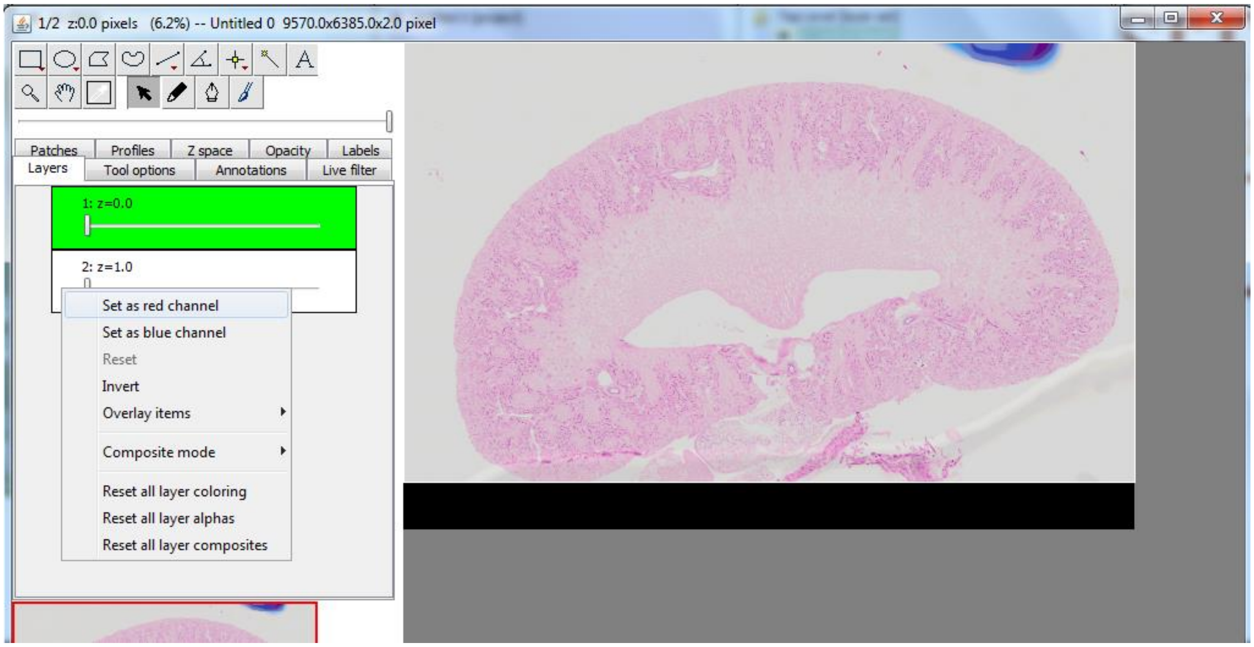

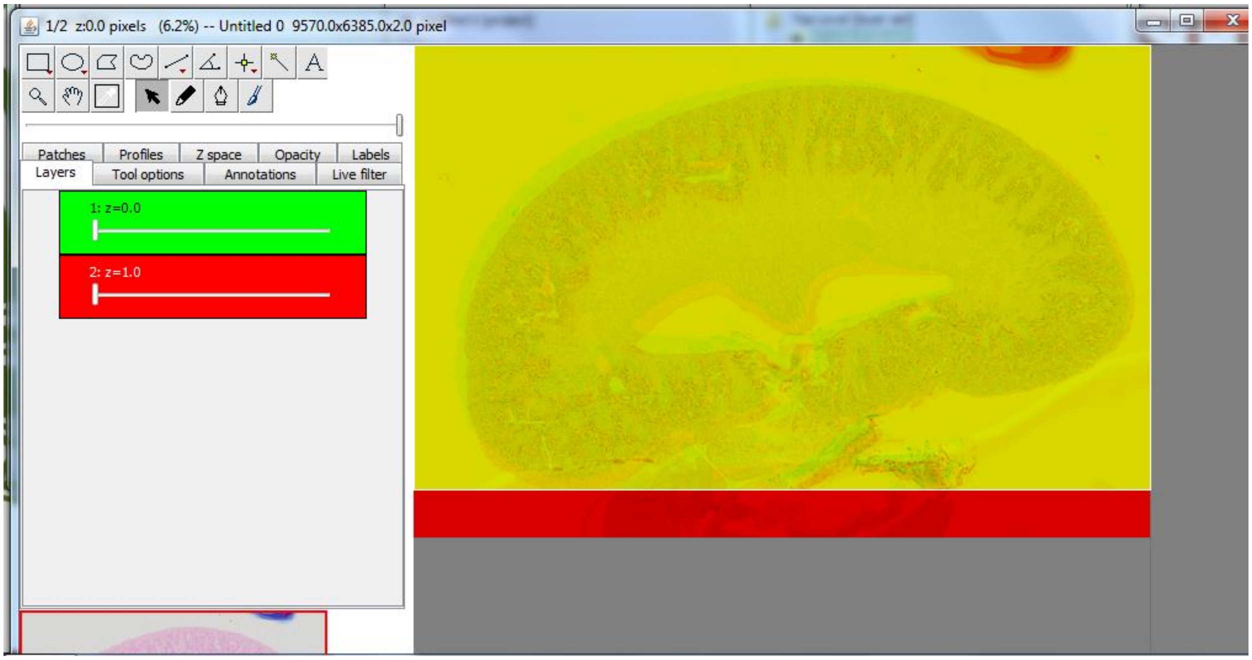

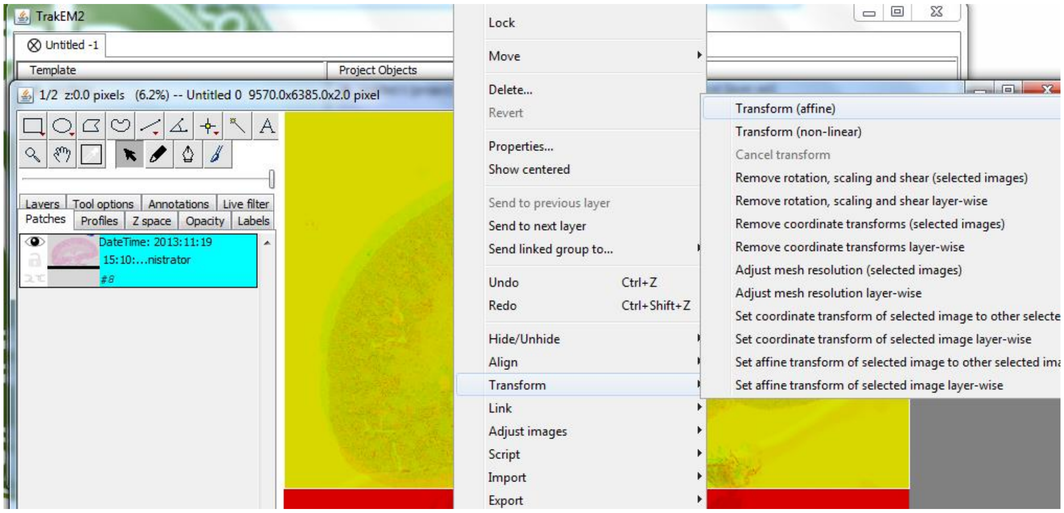
TrakEM2
Untitled -1
Template
Project Objects
1/2 z:0.0 pixels (6.2%) -- Untitled 0 9570.0x6385.0x2.0 pixel
Layers
Tool options
Annotations
Live filter
Patches
Profiles
Z space
Opacity
Labels
DateTime: 2013:11:19
15:10:...nistrator
#8
Lock
Move
Delete...
Revert
Properties...
Show centered
Send to previous layer
Send to next layer
Send linked group to...
Undo Ctrl+Z
Redo Ctrl+Shift+Z
Hide/Unhide
Align
Transform
Link
Adjust images
Script
Import
Export
Transform (affine)
Transform (non-linear)
Cancel transform
Remove rotation, scaling and shear (selected images)
Remove rotation, scaling and shear layer-wise
Remove coordinate transforms (selected images)
Remove coordinate transforms layer-wise
Adjust mesh resolution (selected images)
Adjust mesh resolution layer-wise
Set coordinate transform of selected image to other selecte
Set coordinate transform of selected image layer-wise
Set affine transform of selected image to other selected ima
Set affine transform of selected image layer-wise

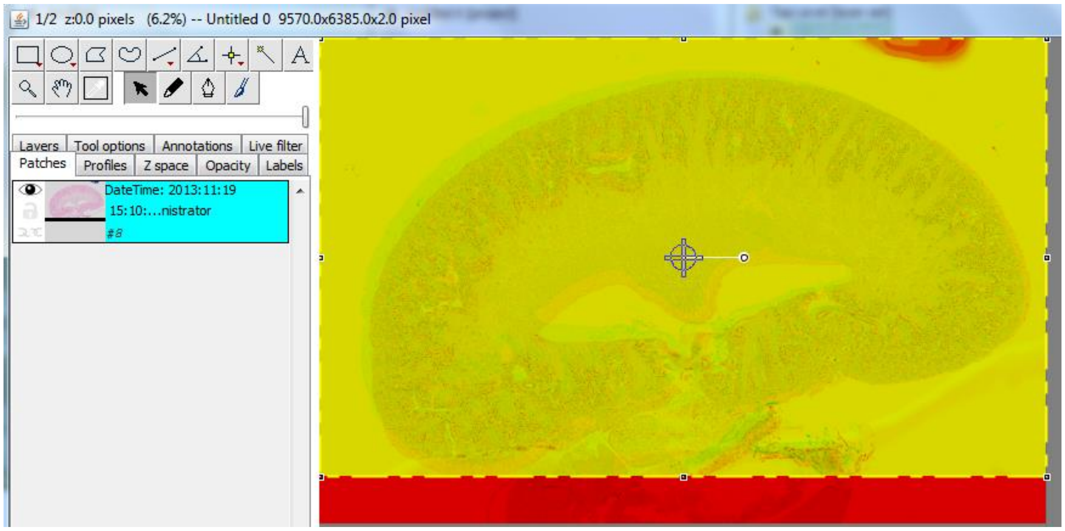
1/2 z:0.0 pixels (6.2%) -- Untitled 0 9570.0x6385.0x2.0 pixel
Layers
Tool options
Annotations
Live filter
Patches
Profiles
Z space
Opacity
Labels
DateTime: 2013:11:19
15:10:...nistrator
#8

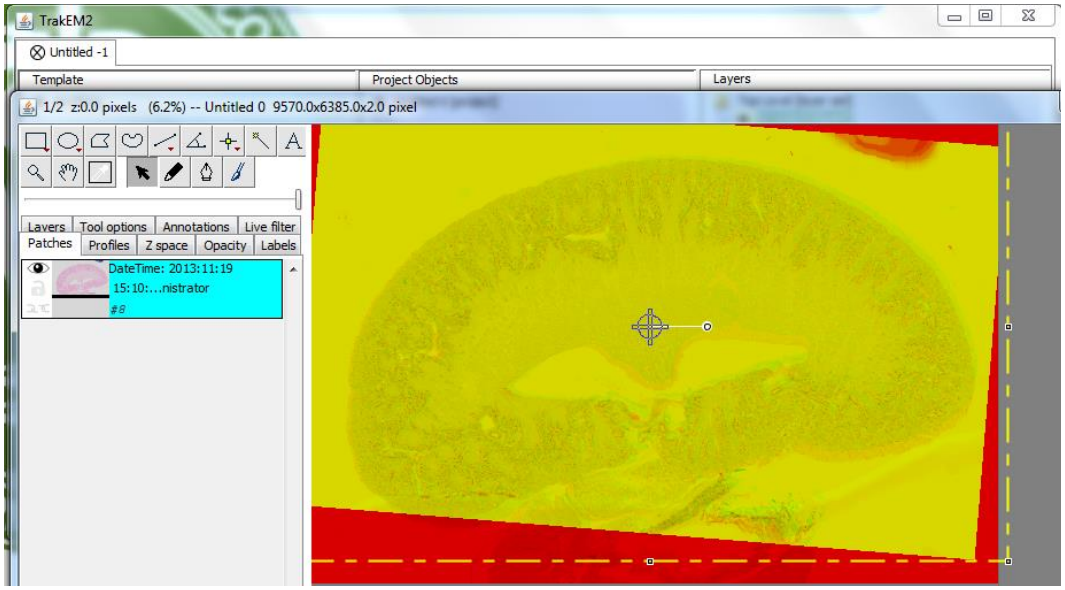
TrakEM2
Untitled -1
Template
Project Objects
Layers
1/2 z:0.0 pixels (6.2%) -- Untitled 0 9570.0x6385.0x2.0 pixel
Layers
Tool options
Annotations
Live filter
Patches
Profiles
Z space
Opacity
Labels
DateTime: 2013:11:19
15:10:...nistrator
#8

6. Identify disector particles, points or other using the disector tool by first selecting it in the "Z space" tab. Be sure to use the PEN tool
    a. Marks in the previous layer appear in red, and marks in the next layer appear in blue and marks in the current layer appear yellow.
    b. Useful keyboard shortcuts in the TrakEM2 environment
        i. <, > used to switch between layers
        ii. -, + to zoom in and zoom out
        iii. With the left mouse button:
            1. Click outside any existing marks to add a new mark, starting a new item to count.
            2. Click on the red or blue ghost of a mark to add a new mark for that item.
            3. Drag to move a mark.
        iv. With the click-wheel you can click and drag to navigate the current image layer or use the "Navigator Panel"

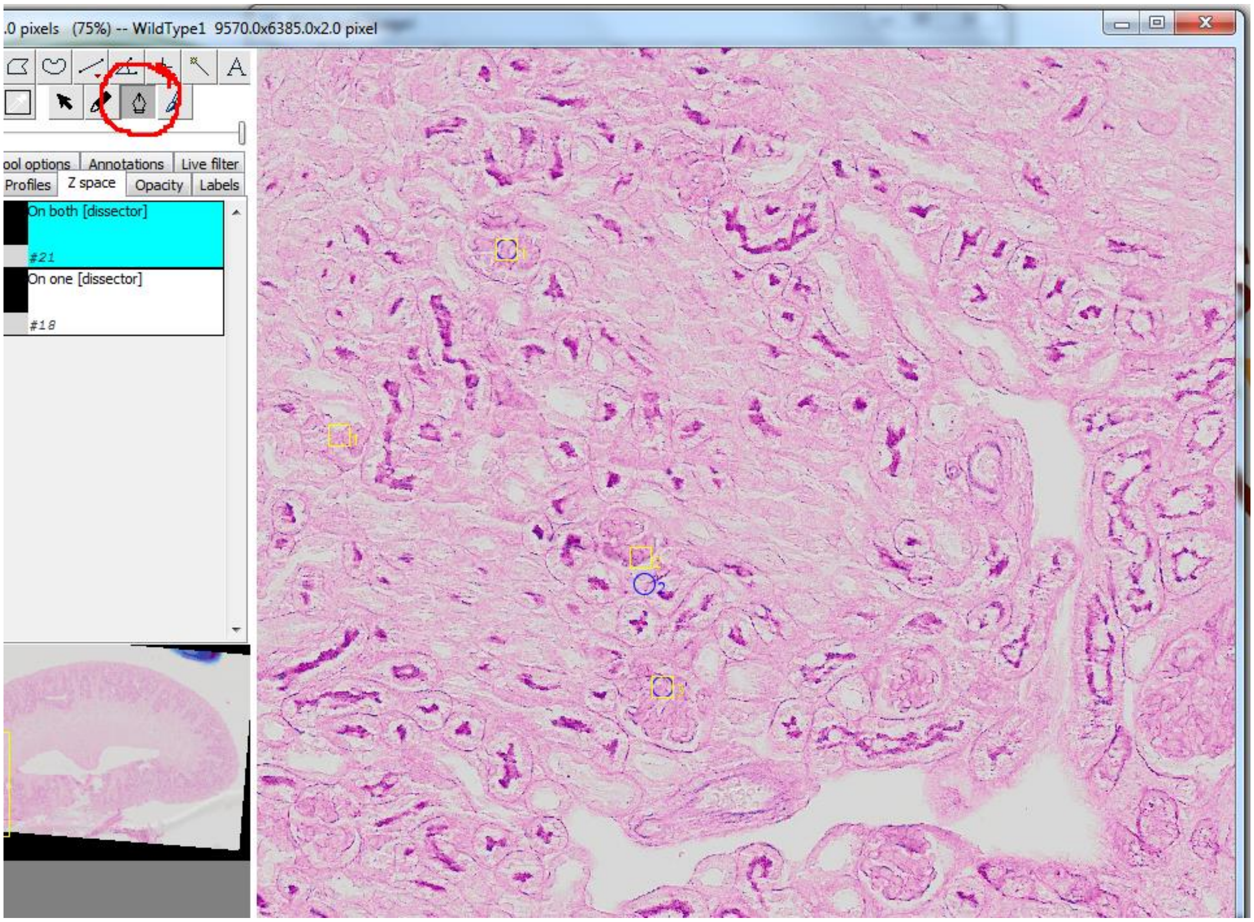

7. Generate reports

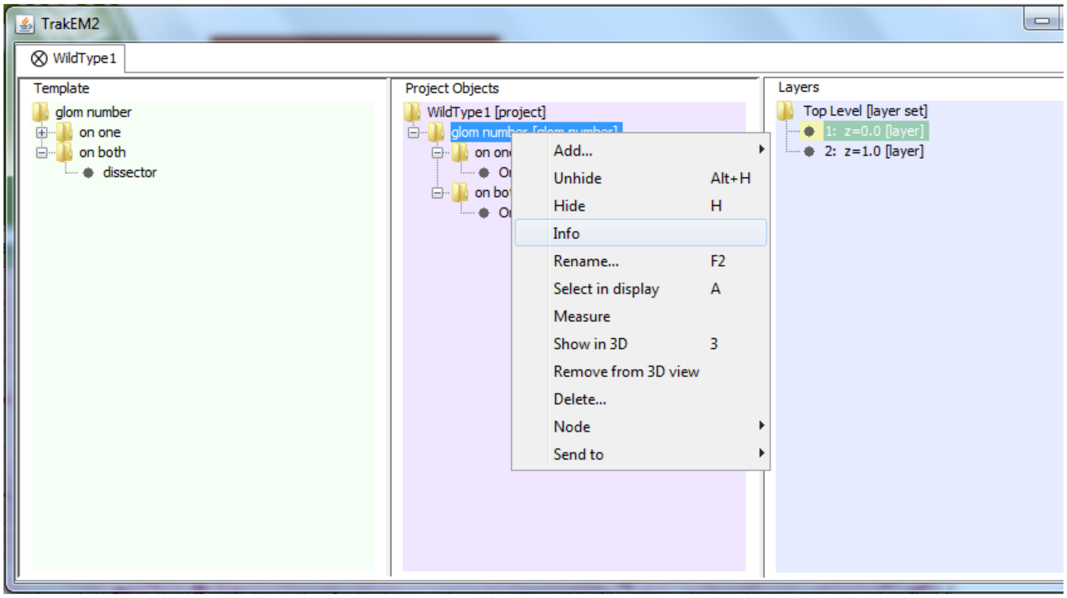

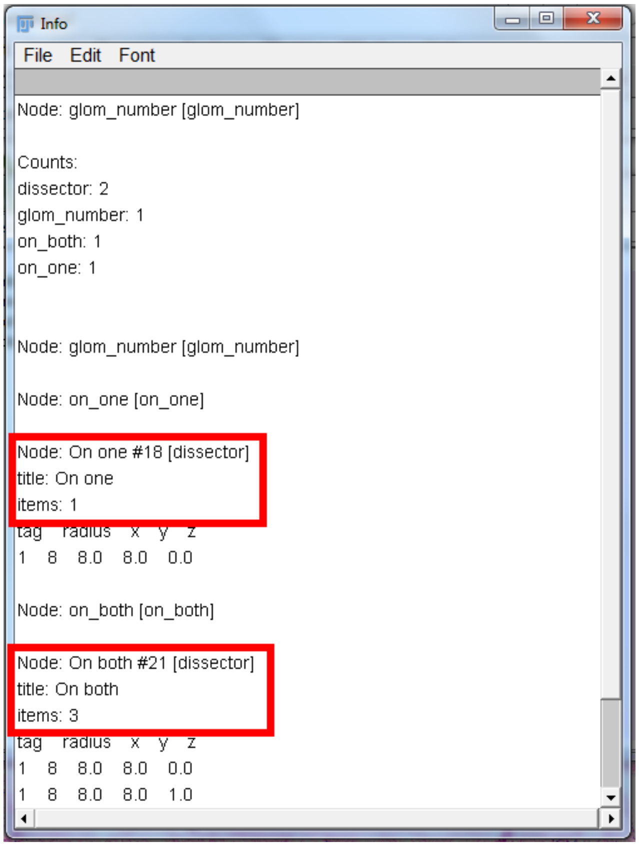

For more details and tutorials see the users manual of TrakEM2
http://www.ini.uzh.ch/~acardona/trakem2_manual.html
